# Critical Review of the Ankle Brachial Index

**DOI:** 10.2174/157340308784245810

**Published:** 2008-05

**Authors:** Tahir H Khan, Falahat A Farooqui, Khusrow Niazi

**Affiliations:** 1 Hospitalist / Instructor of Medicine, Emory University Dept of Medicine, Emory Crawford Long Hospital, 550 Peachtree St, MOT 6th Fl Rm 4307, Atlanta, GA 30308, USA; 2 Dept of Medicine / Div of Cardiology, Emory University, 550 Peachtree St, MOT 6th Floor, Atlanta,GA 30308, USA; 3 Emory University School of Medicine, Director of Peripheral Intervention, Emory Crawford Long Hospital, 550 Peachtree St, MOT 6th Fl Rm 4307, Atlanta, GA 30308, USA

**Keywords:** Peripheral arterial disease, Ankle Brachial index, ABI Sensitivity and Specificity, Atherosclerosis, Cardiovascular morbidity and mortality.

## Abstract

Ankle brachial index (ABI) has been utilized in the management of peripheral arterial disease (PAD).ABI is a surrogate marker of atherosclerosis and recent studies indicate its utility as a predictor of future cardiovascular disease and all-cause mortality. Even so, this critical test is underutilized. The purpose of this review is to summarize available evidence associated with ABI methodology variances, ABI usage in the treatment of PAD, and ABI efficacy in predicting cardiovascular disease. This review further evaluates how ABI is used in the prognosis and follow-up of lower extremity arterial disease.We reviewed the most current American College of Cardiology guidelines for the management of PAD, the Trans Atlantic Intersociety Consensus (TASC) working group recommendations, and searched the Medline for the following words: ankle brachial index, ABI sensitivity and specificity, and peripheral arterial disease.

The ABI is a simple, noninvasive clinical test that should not only be applied to diagnose PAD, but also to provide important prognostic information about future cardiovascular events. Although the ABI has been employed in clinical practice for some time, our review of various studies reveals a lack of standardization regarding both the method of measuring ABI and the cutoff point for abnormal ABI. It is extremely important that we understand all aspects of this crucial test, as it is now being recommended as part of a patient’s routine health risk assessment.

## INTRODUCTION

Peripheral arterial disease (PAD) of the lower extremities is a common disease affecting approximately 12 million people in the United States [1]. Atherosclerosis is the major cause of PAD of lower extremities [2]. The prevalence of PAD varies based on the population surveyed and the methodology of computing the ankle-brachial index (ABI) [3-5].  The ABI is the preferred initial screening test to help diagnose and grade the obstruction of peripheral arterial disease (PAD) in the legs. Interval ABI results are used to monitor the efficacy of revascularization procedures of lower extremities. Additional uses of ABI include predicting the prognosis regarding the limb salvage, wound healing and future cardiovascular related morbidity and mortality [6].

## HISTORICAL PERSPECTIVE

Arterial measurements in lower extremities were first described by Naumann in 1930 [7].  In 1950,Winsor was first to use ABI measurements in patients with peripheral arterial disease [8].

## METHODS OF MEASURING ANKLE BRACHIAL INDEX

**Fig. (1):**
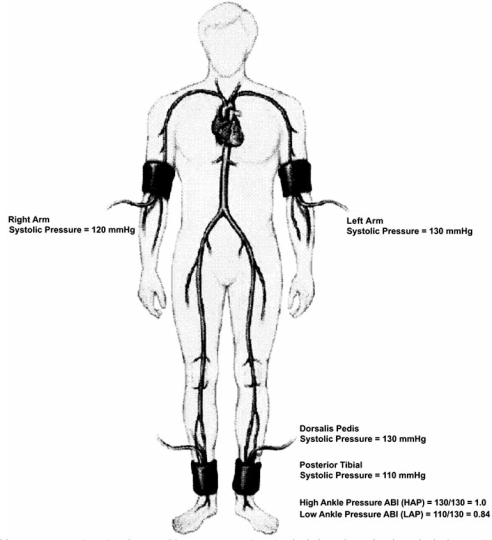
High ankle pressure ABI (HAP) and Low ankle pressure ABI (LAP) calculations shown in a hypothetical case.

The measurement of the ABI involves recording the systolic pressures in the brachial artery at each elbow and systolic pressures in the posterior tibial and the dorsalis pedis arteries at each ankle. The result is reported as a ratio of the ankle systolic pressure in the numerator,over the higher brachial pressure in the denominator. The ABI is calculated for each leg separately, and the lower of the two values is taken as a result for the patient. Numerous methods of calculating the ABI have been described based with variances in the numerator taken in the ABI equation:

- The current method recommended by the ACC/AHA involves using the higher of the two ankle systolic arterial pressures, termed high ankle pressure (HAP) (Fig. **1**) as the numerator in the ABI equation [2,6,9-12].
- A second method reported in the literature uses the lower of the two ankle systolic arterial pressures,termed low ankle pressure (LAP) as the numerator when calculating the ABI [5,13,14].
- A calculation applied in some epidemiological studies uses the average of the two ankle systolic pressures as the numerator in the ABI equation [15,16].
- A few studies have used the posterior tibial artery systolic pressure to calculate ABI [17,18].

## INTERPRETATION OF ABI RESULTS

The patient is diagnosed with PAD when the ABI is ≤ 0.9 [2,19]. PAD is graded as mild to moderate if the ABI is between 0.4 and 0.9, and an ABI less than 0.40 is suggestive of severe PAD [19]. An ABI value greater than 1.3 is also considered abnormal, suggestive of non-compressible vessels.

## CORRELATION OF ABI WITH LOWER EXTREMITY FUNCTION AND OUTCOMES

Patients with mild to moderate PAD are likely to experience lower extremity pain with exercise (claudication). Patients with ABI less or equal to 0.5 are likely to have lower extremity pain while resting [20]. Criqui *et al.* reported that the incidence of claudication (applying Rose criteria) was 49 % in patients with ABI < 0.6 compared to 34 % in patients with ABI between 0.6 and  < 0.9 [21].

Several specific ABI values indicate evidence of correlation with the leg function in multiple studies. In a study of 865 women aged 65 and older, McDermott *et al.* discovered that subjects with ABI < 0.6 were at higher risk of having impaired walking abilities [13]. In an earlier study McDermott *et al.* [22] reported that among patients with PAD, decreases in ABI values are associated with decreases in six minute walk distance, maximum walking speed, and walking endurance. Each increase of 0.4 in the ABI was associated with an increase in the six minute walk distance and usual and maximal walking speed.

An ABI <0.4 increases the risk of limb loss, gangrene, ulceration and delayed wound healing [6,23]. However, an absolute ankle systolic pressure less than 60 mmHg, rather than the ABI value, has been found to correlate better in terms of viability of the lower extremities in PAD [2,24].

## SENSITIVITY AND SPECIFICITY OF THE ABI IN THE DIAGNOSIS OF PAD

Numerous studies have reported that the ABI, when compared to angiography, has a sensitivity of more than 90% and a specificity of more than 95% in diagnosing 50% stenosis of the lower extremity arteries [6,11,12,16,17,25,26]. However, Schroder *et al.* recently reported that the HAP ABI had a sensitivity of 68% and a specificity of 99% [27]. The authors reported the LAP ABI sensitivity and specificity to be 89 and 93% respectively. Niazi *et al.* reported that the HAP ABI had a sensitivity of 69% with a specificity of 83%. The sensitivity and specificity of the LAP ABI was 84% and 64% respectively [28].

Feigelson *et al.* [29] evaluated the sensitivity of an ABI < 0.8 to be 39 % within the entire cohort and 70% in patients with PAD (This study is reported by ACC/AHA guidelines that ABI has a sensitivity of 89% in diagnosing PAD).  Lijmer *et al.* has reported that an ABI value of 0.91 had a sensitivity of 79% and a specificity of 96% to detect 50% or more stenosis of lower extremity arteries defined on angiography [30]. Angiograms of the lower extremities were available for only 53 patients (12% of the total patients in study). Stoffers *et al.* applied ROC analysis and reported the sensitivity and specificity for ABI  < 0.97 to be 79% and 82% respectively [31]. When two vascular technicians using ultrasound technology interpreted the ankle arterial pulse wave forms as “pathologic”, the diagnosis of PAD was determined. No angiographic confirmation for the diagnosis of PAD was provided.

Based on the evaluated studies, we find that the lower ankle pressure ABI has better sensitivity in diagnosing PAD. Additionally, the context in which few studies [24,32-34] are reported (that ABI has been shown to be > 90% sensitive and > 95% specific to diagnose 50% stenosis of lower arteries) is inaccurate. Carter and Yao [32,33] only concluded that ABI was low in patients with PAD without providing any ABI sensitivities. Ouriel did report in his two studies [23,34] that ABI  < 0.97 has a sensitivity of more than 97% and specificity of more than 100% respectively in diagnosing PAD. Nevertheless, the lack of the authors definition of PAD in terms of diameter stenosis of the arteries weakens the validation of their findings. 

## ROLE OF THE ABI IN THE FOLLOW UP OF PAD PATIENTS AFTER LOWER EXTREMITY INTERVENTION

Mclafferty *et al.* reported the sensitivity and specificity of ABI to detect the progression of lower extremity arterial disease after surgical intervention [35].  One hundred fourteen patients (193 Limbs) were followed for a mean of 3.3 years. A baseline ABI was established for each patient, followed by another ABI within a week of the revascularization procedure. Progression of the PAD was documented with arterial duplex scanning or arteriography of the lower extremities if clinically indicated. Disease progression was only monitored in native arteries and not in the bypass grafts. The authors reported that a drop of the ABI more than 0.15 carried a sensitivity of 41% and a specificity of 84% to detect the progression of lower extremity arterial disease.

Decrinis *et al.* tested the sensitivity and specificity of ABI in patients who had undergone angioplasty of the superficial femoral artery stenosis [36]. ABI were performed postoperative day one, three, six and twelve months after the procedure. Follow up angiograms of the lower extremities were performed one year after angioplasty. A total of 116 patients were enrolled in the study. (Angiographic definition of re-stenosis was progression of post PTA 50% stenosis to more than 70% at follow up angiogram or an increase in the stenosis severity to 10 % or more of predilation obstruction.) For an ABI drop of 0.10 the sensitivity and specificity were 72% and 82% respectively in predicting restenosis using their criteria. An ABI drop of 0.15 had a sensitivity and specificity of 66% and 100% respectively for restenosis. In predicting the patency of the post PTA vessel, an improvement of ABI of 0.10 post PTA had sensitivity and specificity of 79 % and 92%. ABI improvement of 0.15 post PTA had sensitivity and specificity of 67% and 100% respectively for patency of post PTA artery. 

## ABNORMAL ABI AS MARKER OF ATHEROSCLEROSIS AND PREDICTOR OF FUTURE CARDIOVASCULAR MORBIDITY AND MORTALITY

The unique role that ABI plays as a marker of atherosclerosis is clear by its correlation with cardiovascular disease (CVD) in multiple population based studies [9-18].  The measurement of ABI has been recommended as part of risk assessment and primary prevention of CVD in asymptomatic individuals who have intermediate risk factors for CVD [37,38].  There is consensus that an abnormal ABI in an otherwise asymptomatic individual would categorize him in the high risk category for future CVD. The American Diabetes Association recommends routinely screening all patients with diabetes above age 50 and in all diabetics with risk factors (e.g. smoking, hyperlipidemia etc) for PAD under age 50 [39].

### 1 Cardiovascular Disease (CVD) and Mortality

Numerous epidemiologic studies have reported up to four fold increased rates of cardiovascular disease and mortality with abnormal ABI [9-18,40-49]. Findings of some of these are summarized in Table **1**.

Vogt *et al.* reported that the mortality rates from atherosclerotic heart disease doubled (RR 2.0 95% CI 1.4-2.9) with each 0.5 units drop in the ABI [16].  The ten year mortality rates of CVD in patients with an ABI < 0.5 was 37%, compared to a 27 % in patients with ABI values ranging between 0.5- 0.7, 22 % in patients with ABI 0.7- 0.9 and 17 % in patients with ABI values >0.9(P=0.0039). Relative risk for all cause mortality was higher (1.95, 95% CI 1.42-2.68) in subjects with ABI < 0.5 compared to subjects with ABI values ranging between 0.51-0.7 (RR 1.59, 95% CI 1.18-2.15).

O’Hare *et al.* investigated the rates of cardiovascular disease and mortality across different levels of ABI [40]. The cohort included 5748 subjects, their mean age was 73 years, and the mean follow-up was 11 years. The study found that subjects with an ABI < 0.6 was consistently associated with increased CV mortality (hazard ratio [HR] 2.13 95%,CI 1.49-30.5), all cause mortality (HR 1.82,95%CI 1.42-2.32) and cardiovascular events(HR 1.60,95% CI 1.09-2.34) compared to participants with ABI 1.10-1.20. In the Framingham Offspring study the prevalence of CAD was 30% in patients with an ABI  < 0.9 compared to 10% in subjects with ABI >1.0 (P 0.001) [41]. Among the participants of the HOPE trial, the percent rates of CV disease, CV death and all cause mortality in subjects with an ABI >0.9 were 10.1,5.3 and 8.8 respectively, compared to 13.7, 8.6,12.8 in the ABI group 0.6-0.9, and 13.4, 9.4 and 14.7 in the ABI group < 0.6 respectively [42]. 

### 2 Risk for Stroke / Transient Ischemic Attack (TIA)

Zheng *et al.* reported that the risk of Stroke /TIA for men with ABI < 0.9 was four times (Odds Ratio 4.2 -4.9, 95% CI 1.8-9.5) than in those with an ABI >0.9 [43]. In the Framingham study, the risk of stroke/ TIA was two fold (Hazards ratio 2.0, 95% CI 1.1-3.7) among participants with an ABI< 0.9 [15].  In the Framingham offspring study the prevalence of stoke was 9% in men with ABI< 0.9 compared to 2 % in men with ABI > 1.0 (0.001) [41]. In the Cardiovascular Health Study, patients with ABI <0.8 had twice the rates of stroke/TIA [44].  Similarly, Leng *et al.* reported that subjects with an ABI <0.9 had increased risk of stroke(RR 1.98, 95% CI 1.05-3.77) [45].

In a meta analysis of nine studies Doobay reported the predictive value of ABI in predicting future cardiovascular events [46]. The authors reported that an ABI less than 0.9 has a sensitivity and specificity of 16.55 and 92.7% for CAD (likelihood ratio 2.53), 16.0% and 92.2% for stroke (likelihood ratio 2.45) and 41.0% and 87.9% for cardiovascular mortality (likelihood ratio 5.61), respectively. 

Heald *et al.* performed a systematic review of 11 published studies with a combined number of 44590 subjects [47]. The authors reported that ABI  < 0.9 was associated with increased all cause mortality (RR 2.35, P< 0.001), cardiovascular and cerebrovascular mortality (RR 2.34, P =0.002), fatal and non-fatal coronary heart disease (RR 2.13, P= 0.003) and fatal and non-fatal stroke (RR 1.86, P=0.07). 

An ABI < 0.9 identifies subjects with increased future cardiovascular morbidity and mortality. However, across the range of ABI a value < 0.6 has been found with increased CV events (Table **1**). 

### 3 Abnormal ABI as Predictor of Morbidity and Mortality in Post-Coronary Artery Bypass Graft Patients

Abnormal ABI has been found as prognostic indicator for survival and complications among patients undergoing coronary artery bypass surgery. Aboyans V *et al.* studied the role of abnormal ABI as a marker for long term prognosis in the post coronary artery bypass graft patients (CABG) [48]. The mean age of the patients was 69 years with a follow-up of 4.4 years. Patients with clinical PAD (defined as history of vascular surgery and/or history of intermittent claudication) and sub clinical PAD (defined as ABI<0.85) had three fold excess risk of primary end points (composite of cardiovascular death, non-fatal acute coronary syndrome, non-fatal stroke, TIA and coronary or peripheral revascularization) compared to patients without PAD after CABG. Risk of acute coronary syndrome was more than two times (HR 2.12 to 2.35) in patients with clinical and sub clinical PAD.

Burek *et al.* studied the five year mortality rates in Post CABG/ Coronary PTCA patients. The five year mortality rate was 14 % in patients with ABI  < 0.9 compared to 3% in patients with ABI > 0.9 (RR 4.9 95% CI 1.8-13.4, P 0.001) [49]. The investigators reported that abnormal ABI< 0.9 was a strong predictor of mortality among patients with multivessel CAD.

## CONCLUSION

We conclude that ABI is the screening test of choice for the diagnosis of patients with PAD due its simplicity, reproducibility and cost effectiveness. Recent studies suggest the use of low ankle pressure ABI as the method for calculating the ABI due to its better sensitivity. Use of ABI is recommended as part of management of patients who have undergone lower extremity revascularization procedures.

As surrogate marker for atherosclerosis, ABI has been found to give very important prognostic information regarding future cardiovascular events. We notice different methods and cutoff points for abnormal ABI have been used in different epidemiologic studies. There is a need for a uniform method of ABI to be used in studies. Current evidence suggests the use of ABI for identifying high risk patients for future cardiovascular and cerebrovascular mortality. In doing so, one can aggressively modify risk factors to prevent both short and long term events.

## Figures and Tables

**Table 1:** Cardiovascular, Stroke / TIA and All Cause Mortality Across Range of ABI in Published Studies

Study	No Study Subjects	ABI	Effect Measure	CVD	CVD Mortality	All Cause Mortality	Stroke/ TIA

Diehm *et al.* 12	6880	< 0.9	Odds Ratio	1.53	N/A	N/A	1.77

Vogt *et al.* 16	1930	>0.9	Relative	N/A	RR 2.0 each	1.0	No Significant association
		<0.9-0.71	Risk		< 0.5 Drop in	1.15-0.95	
		<0.7-0.51			ABI.	1.59-1.70	
		<0.50				1.95-2.13	

O’Hare *et al*. 40	5748	0.91-1.0	Hazard	1.37	1.60	1.40	Included in CVD.
		0.81-0.90	Ratio	1.72	2.37	1.73	
		0.71-0.81		1.63	2.01	1.80	
		0.61-0.70		1.57	2.31	2.08	
		<0.61		1.60	2.13	1.82	

Ostergen *et al.* 42	8986	> 0.9	Four year	10.1	5.3	8.8	3.5
		0.9-0.6	Clinical outcomes	13.7	8.6	12.8	4.3
		<0.6		13.4	9.4	14.7	5.9
			(Percent rates).				
				P 0.0038	P <0.0001	P .0002	P 0.234

Zheng *et al.* 43	15106	<0.9	Odds Ratio	4.5	N/A	N/A	4.3
				(P<0.05)			(P <.001)
							
							
		0.91-1.0		2.4			1.7

Leng *et al*. 45	1592	1.0-0.9	Five Year	5%	6%	11%	3%
		0.9-0.71	Incidence	7%	8%	16%	3%
		<0.7		9%	21%	34%	3%
				P.057	P <.001	P <.001	P .020

N/A: Data not provided; CVD: Cardiovascular Disease; TIA: Transient Ischemic attack.
